# Space-Time-Coding Digital Metasurfaces: Principles and Applications

**DOI:** 10.34133/2021/9802673

**Published:** 2021-05-22

**Authors:** Lei Zhang, Tie Jun Cui

**Affiliations:** ^1^State Key Laboratory of Millimeter Waves, Southeast University, Nanjing 210096, China; ^2^Institute of Electromagnetic Space, Southeast University, Nanjing 210096, China; ^3^Center for Intelligent Metamaterials, Pazhou Laboratory, Guangzhou 510330, China

## Abstract

Space-time-modulated metastructures characterized by spatiotemporally varying properties have recently attracted great interest and become one of the most fascinating and promising research fields. In the meantime, space-time-coding digital metasurfaces with inherently programmable natures emerge as powerful and versatile platforms for implementing the spatiotemporal modulations, which have been successfully realized and used to manipulate the electromagnetic waves in both the spectral and spatial domains. In this article, we systematically introduce the general concepts and working principles of space-time-coding digital metasurfaces and provide a comprehensive survey of recent advances and representative applications in this field. Specifically, we illustrate the examples of complicated wave manipulations, including harmonic beam control and programmable nonreciprocal effect. The fascinating strategy of space-time-coding opens the door to exciting scenarios for information systems, with abundant applications ranging from wireless communications to imaging and radars. We summarize this review by presenting the perspectives on the existing challenges and future directions in this fast-growing research field.

## 1. Introduction

Metastructures known as three-dimensional (3D) metamaterials [1–8] and two-dimensional (2D) metasurfaces [9–11] are constructed by periodically or aperiodically arranging subwavelength meta-atoms that can be artificially engineered, which have undergone fast developments in the past 20 years and widely been used to control the electromagnetic (EM) waves in extraordinary ways, leading to numerous fascinating phenomena, novel devices, and exciting applications. Compared with the 3D bulky metamaterials, 2D metasurfaces have the superiorities of low loss, ultrathin thickness, and simple fabrication and have been steadily attracting increasing research interests [12–25]. In particular, the generalized Snell's laws were put forward by Yu et al. in 2011 [12], which greatly boost the development of metasurfaces. This type of metasurfaces, governed by the generalized Snell's law, exhibits spatially gradient phase discontinuities along the interfaces, which have been applied to manipulate the EM wavefronts from the microwave region to the visible light. However, the conventional space gradient metasurfaces do not explore the temporal dimension and are constrained by the Lorentz reciprocity.

In recent years, time-varying and space-time-modulated metastructures have attracted great attention and become one of the most promising research fields [26–56]. Time-varying metastructures provide the degree of freedom to modulate their constitutive parameters in the time domain. Combined with spatial modulations, the space-time-modulated metastructures are characterized by spatially and temporally variant constitutive parameters, such as the permittivity [30–33], conductivity [34–37], and surface impedance [38, 39]. The space-time-modulated metastructures have been studied extensively to produce many novel physical phenomena and interesting applications, including optical isolators [40, 41], breaking the Lorentz reciprocity [30–32, 35, 38, 39, 42–46], Doppler cloaks [47], harmonic generations [36, 48, 49], nonreciprocal antennas [34], full-duplex systems [50, 51], and frequency conversion [52–56]. It is worth mentioning that all these characterization methods introduced in the space-time-modulated metastructures can be regarded as *analog modulation* with continuous variation of parameters [30–56]. Most of these analog-type space-time-modulated metastructures are based on theoretical and/or numerical investigations, whereas experimental realizations remain limited to some extent.

As an emerging branch of metasurfaces, the idea of digital coding metasurface was first put forward by Cui et al. in 2014 [57]. The basic idea underpinning such metasurfaces relies on a finite number of coding elements and yet the ability to realize complicated EM field manipulations. For example, in the simplest binary case, by constructing two elements with opposite reflection/transmission phase as digital bits “0” and “1,” digital coding metasurfaces can achieve wave manipulations by altering the coding sequences in a discretized manner, which greatly simplify the design, optimization, and fabrication process [58–74]. This digital coding representation introduced in such metasurfaces can be regarded as *digital modulation* with discrete coding sequences, which can be easily realized in practice with simple hardware and is naturally suitable for integrating active devices such as diodes. In this way, by independently controlling the active devices embedded in the digital coding elements via a field-programmable gate array (FPGA), digitally programmable metasurfaces can be realized to dynamically control the EM fields and switch among different functions in real time in programmable ways [57, 75–88]. This fascinating concept has been successfully applied to numerous applications from microwaves to terahertz and even acoustics, including reflect/transmit arrays [63–68], beam/polarization manipulations [69, 73, 74], reprogrammable holograms [80], microwave imaging [81, 82], scattering control [70–72], nonlinearity [62, 86], nonreciprocity [87, 88], information processing [89–92], direct transmission of the digital messages [93, 94], and wireless communications [95–100]. Most crucially, digital coding and programmable metasurfaces have built a broad way between the physical world (wave physics) and the digital world (information science), leading to a grand vision of information metasurfaces [101–104]. The reprogrammable character of the information metasurfaces can be further leveraged to perform software-defined [105, 106], self-adaptive [107], and even cognitive functionalities empowered by the artificial intelligence algorithms [81, 82, 108].

In the initial studies, digital coding is defined in the spatial domain and fixed in the temporal domain, in which case the space-domain-coding (SDC) digital metasurfaces can only manipulate the spatial distribution of EM waves [57–107], such as the far-field scattered beams and the near-field patterns. By extending the digital coding from the spatial domain to the temporal domain, time-domain-coding (TDC) digital metasurfaces can control the spectral distribution of reflected waves by dynamically switching the time-coding sequences [109]. In 2018, the general theory of space-time-coding (STC) digital metasurfaces was originally put forward by Zhang et al. [110]. The constitutive parameters (e.g., reflection phases) of the STC digital metasurfaces are jointly encoded in space and time, which can control the EM waves in both the spectral and spatial domains. That is to say, one can simultaneously manipulate the harmonic distribution and propagation direction of reflected waves [110]. The STC digital metasurfaces extend and generalize the concepts of “phase-switched screens” [111] and “time-modulated arrays” [112] and have been applied successfully to harmonic beam control [110], scattering reduction [110], nonreciprocal effect [113], multibit phase generation [114], terahertz harmonic manipulations [115], spread-spectrum camouflaging [116], analog computing [117], and wireless communications [118].

In this article, firstly, we briefly introduce the general concepts and working principles of the SDC and STC digital metasurfaces (Section 2). Subsequently, we present some recent advances and representative applications of the STC digital metasurfaces (Section 3). Finally, we discuss the perspectives on the existing challenges and future research directions of the STC digital metasurfaces in the conclusion part (Section 4).

## 2. General Concept and Working Principles of STC Digital Metasurfaces

**Figure 1:**
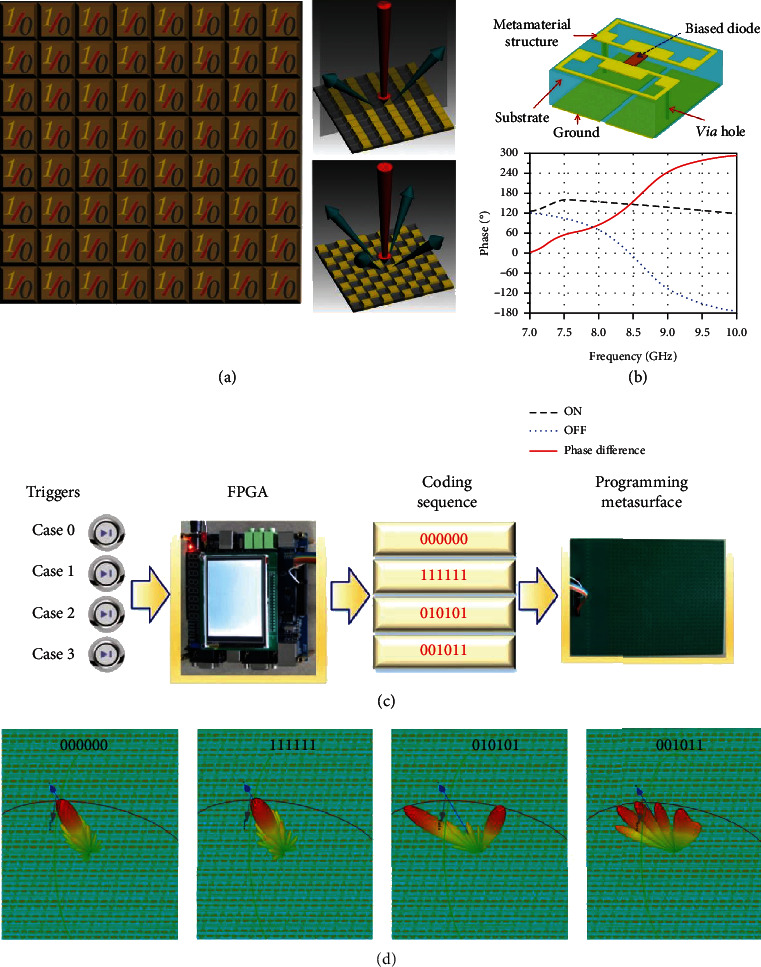
Digital coding and programmable metasurfaces. Reproduced from [57]. (a) Schematic of the digital coding metasurface containing two types of 1-bit elements “0” and “1,” and it scattered beams under different coding patterns. (b) Geometry of 1-bit programmable elements and the corresponding reflection phases. (c) The flow chart of the 1-bit programmable metasurface under the control of an FPGA hardware. (d) Numerically simulated results of scattering patterns for the 1-bit programmable metasurface under different coding sequences.

In this section, we briefly introduce the fundamental concept of digital coding and programmable metasurfaces [57].  Figure 1(a) displays the conceptual schematic of a 1-bit digital coding metasurface, which is composed of two distinct coding elements “0” and “1” with a phase difference of *π*. It can be seen that the digital coding metasurfaces can regulate the reflected EM beams by designing different coding patterns. By embedding active devices such as diodes into the coding elements, programmable metasurfaces are further developed to control the EM fields in real time.  Figure 1(b) shows the geometry and reflection phases of the 1-bit programmable element, which exhibits coding states “0” and “1” when the PIN diode is switched between “OFF” and “ON,” respectively. The coding states of programmable metasurfaces can certainly be extended from 1 bit to multibits. For example, a 2-bit programmable metasurface can be realized by integrating more PIN diodes into each coding element and thereby has four coding states “00,” “01,” “10,” and “11” with the discrete phases of 0, *π*/2, *π*, and 3*π*/2, respectively, and higher-bit programmable metasurfaces can be similarly characterized and realized.  Figure 1(c) shows the flow diagram of the 1-bit programmable metasurface controlled by an FPGA, which can attain different scattered beams by altering different coding sequences of “000000,” “111111,” “010101”, and “001011,” as depicted in Figure 1(d).

**Figure 2:**
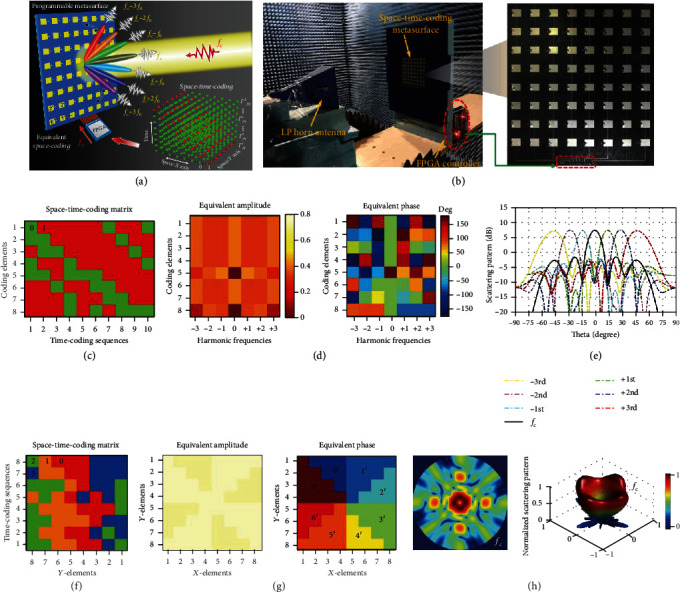
Space-time-coding digital metasurfaces and their applications in harmonic beam steering and shaping. Reproduced from [110]. (a) Conceptual illustration of a digital coding metasurface, which can simultaneously control the spectral and spatial distributions of EM waves. (b) Photo of the experimental setup and the fabricated prototype. (c) Optimized 2D STC matrix for harmonic beam steering. (d) Equivalent amplitudes and phases for the optimized matrix in (c). (e) The corresponding 1D simulated scattering pattern at various frequencies. (f) Optimized 2D STC matrix for beam shaping. (g) Equivalent amplitudes and phases at the central frequency for the optimized matrix in (f), exhibiting an equivalent 3-bit spiral phase distribution. (h) The corresponding 2D and 3D simulated scattering patterns of vortex beam generation.

Next, we present the general concept and working principles of STC digital metasurfaces. Referring to Ref. [110] for details, the STC digital metasurface usually encompasses a 2D array of *M* × *N* programmable elements, which is schematically illustrated in Figure 2(a). The programmable elements represented by yellow patches have identical structures, and each of them is integrated with active devices such as positive-intrinsic-negative (PIN) diodes. By loading different biasing voltages to the active devices, reflection coefficients of programmable elements can be dynamically tailored with a set of quantized amplitudes or phases. For the 1-bit phase-encoding case, the response of each element is switched between two coding states, namely, digital bits “0” and “1” corresponding to the in-phase and out-of-phase reflections, respectively. We assume that the coding state is electronically switched in both the spatial and temporal domains via an FPGA, in accordance with the 3D STC matrix represented by red and green dots in Figure 2(a), in which each element is periodically time-modulated with a set of time-coding sequences. Combined with space modulation, the STC digital metasurface can simultaneously manipulate the spectral (harmonic distribution) and spatial (propagation direction) characteristics of the scattered waves.

As previously assumed in [110], an adiabatic approximate model was put forward to represent the scattered fields of STC digital metasurfaces under the incidence of a normal plane wave with the time-harmonic dependence exp(*j*2*πf*_c_*t*_c_). This analytical model depends on the physical optics approximation that was developed for conventional SDC metasurfaces [57]. By assuming that the time-modulated frequency *f*_0_ is much smaller than the EM carrier frequency *f*_c_, the far-field scattering pattern of the STC digital metasurface in the time domain is approximately written as

(1) $$\begin{array}{c} f\left(\theta ,\varphi ,t\right)=E\left(\theta ,\varphi \right)\overset{N}{\underset{q=1}{\sum }}\overset{M}{\underset{p=1}{\sum }}\Gamma_{pq}\left(t\right)\exp\left(j\frac{2\pi \sin\theta }{\lambda_{\text{c}}}\left[\left(p-1\right)d_{x}\cos\varphi +\left(q-1\right)d_{y}\sin\varphi \right]\right), \end{array}$$

where *E*(*θ*, *φ*) is the far-field scattering pattern of coding elements at the central frequency *f*_c_; *λ*_c_ = *c*/*f*_c_ is the central operating wavelength (with *c* denoting the speed of light in vacuum); *θ* and *φ* are the elevation and azimuth angles, respectively; and *d*_*x*_ and *d*_*y*_ represent the element spacing. Moreover, Γ_*pq*_(*t*) represents the time-varying reflection coefficient of the (*p*, *q*)^th^ element, which is assumed to be a function with the period *T*_0_ and defined as a linear superposition of pulse functions over one period:

(2) $$\begin{array}{c} \Gamma_{pq}\left(t\right)=\overset{L}{\underset{n=1}{\sum }}\Gamma_{pq}^{n}U_{pq}^{n}\left(t\right),\;0<t<T_{0}, \end{array}$$

where *U*_*pq*_^*n*^(*t*) is a shifted rectangular pulse function and defined in one period as

(3) $$\begin{array}{c} U_{pq}^{n}\left(t\right)=\left\{\begin{array}{cc} 1, & \left(n-1\right)\tau \leq t\leq n\tau ,\\ 0, & \text{otherwise}, \end{array}\right. \end{array}$$

where *L* denotes the length of the time-coding sequence, *τ* = *T*_0_/*L* denotes the pulse width of *U*_*pq*_^*n*^(*t*), and Γ_*pq*_^*n*^ = *A*_*pq*_^*n*^exp(*jφ*_*pq*_^*n*^) denotes the reflection coefficient of the (*p*, *q*)^th^ coding element at the central frequency *f*_c_ within the interval (*n* − 1)*τ* ≤ *t* ≤ *nτ*, with *φ*_*pq*_^*n*^ and *A*_*pq*_^*n*^ representing the phase and amplitude, respectively. By decomposing the periodic function Γ_*pq*_(*t*) into the Fourier series Γ_*pq*_(*t*) = ∑_*m*=−∞_^+∞^*a*_*pq*_^*m*^exp(*j*2*πmf*_0_*t*), the Fourier coefficients *a*_*pq*_^*m*^ can be further expressed as

(4) $$\begin{array}{c} a_{pq}^{m}=\overset{L}{\underset{n=1}{\sum }}\frac{\Gamma_{pq}^{n}}{L}\mathrm{sinc}\left(\frac{\pi m}{L}\right)\exp\left(\frac{-j\pi m\left(2n-1\right)}{L}\right). \end{array}$$

By substituting equation (4) into equation (1), the frequency-domain far-field pattern of the STC digital metasurface at the harmonic frequency *f*_c_ + *mf*_0_ can be further expressed as

(5) $$\begin{array}{c} F_{m}\left(\theta ,\varphi \right)=E\left(\theta ,\varphi \right)\overset{N}{\underset{q=1}{\sum }}\overset{M}{\underset{p=1}{\sum }}a_{pq}^{m}\exp\left(j\frac{2\pi \sin\theta }{\lambda_{\text{c}}}\left[\left(p-1\right)d_{x}\cos\varphi +\left(q-1\right)d_{y}\sin\varphi \right]\right). \end{array}$$

It is worth mentioning that these complex-valued coefficients *a*_*pq*_^*m*^ in equation (4) can be synthesized to produce *equivalent amplitudes and phases* through linear combinations of the reflection coefficient Γ_*pq*_^*n*^; that is to say, we can individually regulate the equivalent amplitudes and phases of each coding element at a specific frequency by designing Γ_*pq*_^*n*^ in the time-coding sequences. Hence, for an arbitrary 3D STC matrix represented by equation (2), we can calculate the equivalent excitations (via equation (4)) and scattering patterns (via equation (5)) of the STC digital metasurface at any harmonic frequencies of interest.

## 3. Recent Advances and Representative Applications

With the aid of the fundamental theory introduced above, we can utilize the STC digital metasurfaces to simultaneously manipulate EM waves in both the spectral and spatial domains (see the conceptual illustration in Figure 2(a)). Furthermore, by elaborately designing the STC matrix, information encoding and processing can be carried out not only in the spatial domain but also in the temporal domain. In this section, we mainly consider the phase modulation scheme, in which the amplitude *A*_*pq*_^*n*^ of each coding element is uniform and the phase *φ*_*pq*_^*n*^ is switched among different coding states according to the STC matrix. The STC strategy significantly expands the application range of conventional metasurfaces, leading to many promising applications in radars, imaging, and wireless communications [119]. In what follows, we will focus on the recent advances and representative applications of the STC digital metasurfaces.

### 3.1. Harmonic Beam Control

An attractive application inspired by the STC digital metasurfaces is harmonic beam steering, in which different harmonic beams point to different spatial directions [110], as illustrated in Figure 2(a). Actually, a simple time gradient STC matrix (see Figure 2(b) in [110]) can also be used to realize harmonic beam steering. Different equivalent phase gradients that emerge at different harmonic frequencies explain the essence of harmonic beam steering. However, this simple scheme suffers from the issue of imbalanced power distributions. To solve this issue, the binary particle swarm optimization (BPSO) algorithm is exploited to optimize the STC matrix in [110] so as to equalize power levels at different frequencies. In this case, we consider an STC digital metasurface with 8 × 8 half-wavelength elements and the 1-bit time-coding sequences with the length *L* = 10. Since the beam steering is designed in the one-dimensional plane, each column of elements has the same time-coding sequences so that the 3D coding matrix can be simplified to a 2D matrix (see Figure 2(c)). Figure 2(d) shows the corresponding equivalent amplitudes and phases at different frequencies, from which the balanced amplitude and gradient phase distributions are evident. Accordingly, the harmonic scattering patterns are numerically calculated via equation (5) and shown in Figure 2(e), in which the main beams at different frequencies have uniform power levels and point to different directions in space. To experimentally verify this design, a prototype of a 1-bit STC digital metasurface working around the frequency of 10 GHz was manufactured by the printed circuit board (PCB) technology (see [110] for details), as displayed in Figure 2(b). When the PIN diode inserted in the coding element is switched between “ON” and “OFF” states, the reflection coefficient can obtain a phase difference of 180°. For the one-dimensional beam steering, eight coding elements in each column have the same biasing voltages via an FPGA hardware control board, which provides eight control voltages according to the STC matrix in Figure 2(c). In this experiment, the time-modulated frequency is assumed to be *f*_0_ = 0.5 MHz, and the corresponding switching rate of PIN diodes is 5 MHz. The measured far-field patterns agree well with the theoretical predictions, thereby validating the effect of harmonic beam steering.

As can be observed from the above example, only the harmonic beams (*m* ≠ 0) are deviated from the broadside, while the 1-bit STC matrix cannot achieve beam steering at the central frequency (*m* = 0). This limitation essentially stems from equation (4), in which 1-bit time-coding sequences can only generate equivalent phases of 0° and 180° at the center frequency. To relax this restriction, we can adopt the higher-bit encoding schemes (e.g., 2 bits associated with digits “0,” “1,” “2,” and “3”) in the physical metasurface design [110]. Next, we introduce an example of beam shaping, in which programmable elements with 2-bit coding states are used to attain equivalent 3-bit coding (represented by the digits “0′,” “1′,” “2′,” “3′,” “4′,” “5′,” “6′,” and “7′”). In this example, the STC digital metasurface is the same as the previous example, but the length of time-coding sequences is *L* = 8. Figure 2(f) shows eight sets of 2-bit time-coding sequences, which are carefully selected to achieve equivalent 3-bit responses at the center frequency. The programmable metasurface is divided into eight parts accompanied by the rotated distribution of those time-coding sequences. Figure 2(g) shows the corresponding equivalent amplitudes and phases at the center frequency *f*_c_, in which the equivalent 3-bit phases exhibit a spiral-like distribution and can generate a vortex beam with orbital angular momentum (OAM). The corresponding far-field scattering patterns are shown in Figure 2(h), from which a typical hollow-center profile of the vortex beam can be clearly observed. Overall, this STC scheme provides a new pathway to realize multibit programmable metasurfaces (see also the detailed discussion in Section 3.4 below), which gets rid of the complex metasurface layout and control circuit system, and further achieves precise field manipulation in both the spatial and spectral domains.

### 3.2. Reduction of Scattering

With the rapid development of radar and stealth technologies, how to reduce the radar cross-section (RCS) of a target has always been a pivotal issue. So far, many methods have been exploited for RCS reduction. For example, radar-absorbing materials are widely used to absorb the EM energy, whereas low-scattering metasurfaces using the phase cancellation method can disperse the incident waves to many directions in the spatial domain. Besides, the phase-switched screen was proposed to redistribute the scattered power to odd harmonics in the frequency domain [111]. Against this background, another interesting application enabled by the STC digital metasurface is to reduce RCS [110]. This novel STC mechanism can be used to effectively redistribute the scattered energy in both the spectral and spatial domains, which completely differs from the conventional methods and brings out a better performance of RCS reduction.

**Figure 3:**
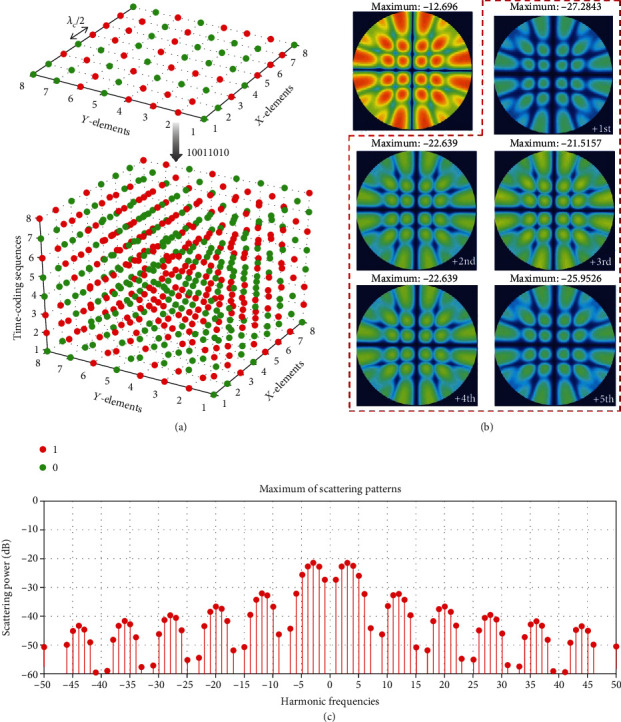
Scattering control via STC digital metasurfaces. Reproduced from [110]. (a) An optimized 2D space-coding matrix and a 3D STC matrix for RCS reduction. (b) The corresponding scattering patterns at various frequencies. (c) Maxima of the scattering patterns from the -50^th^ to the +50^th^ harmonic frequencies, corresponding to the 3D STC matrix in (a).

We first consider a BPSO-optimized space-coding shown at the top of Figure 3(a), its corresponding backscattered power is distributed uniformly in all possible directions in the space domain, and the maximum of backscattered power is reduced by ~12.7 dB by comparison with the reference metallic plate, as shown in the top-left corner of Figure 3(b). And then, we further apply a time-coding sequence “10011010” to this BPSO-optimized space-coding, forming a 3D STC matrix shown at the bottom of Figure 3(a). In this case, the incident power can be redistributed more uniformly in both the spatial and spectral domains, and the maximum of scattered power at different harmonics is further decreased by ~8.82 dB compared with the case of only space-coding in Figure 3(a). The corresponding scattering patterns at several harmonics are shown in the red dashed box in Figure 3(b), and the maximum of scattered power at different harmonics is decreased by ~21.52 dB compared with the reference metallic plate (see Figure 5(f) in [110]). Moreover, Figure 3(c) also shows the maxima of scattering patterns from the -50^th^ to the +50^th^ harmonic frequencies, corresponding to the 3D STC matrix in Figure 3(a). It can be observed that the maximum intensity gradually decreases with the increase of the harmonic order and drops significantly with respect to the first few harmonics. Generally, with the aid of the STC strategy, the scattered energy can be excellently suppressed in both the spatial and spectral domains, which ensures a more effective and robust effect of RCS reduction. Other related applications of scattering control also include spectral camouflage by introducing random time-modulated signals [116].

### 3.3. Programmable Nonreciprocal Effects and Frequency Conversions

In the area of EM engineering, breaking reciprocity has attracted research interests owing to its pivotal role in releasing the reciprocal limitation in communications, energy harvesting, and thermal management. Conventional methods based on nonlinear and magnetic materials can attain nonreciprocal effects but have many disadvantages in practice. Besides, time-variant methods have emerged as appealing alternatives to break reciprocity [30–32, 35, 42], in which space-time-modulated metastructures have aroused great attention [38, 39, 41, 44]. In recent years, some theoretical and experimental works based on metasurfaces have been proposed to achieve nonreciprocal effects by introducing a suitable spatiotemporal phase gradient [45, 46].

More specifically, a 2-bit STC digital metasurface has recently been leveraged to realize the programmable nonreciprocal effect via the time modulation of the phase responses [113], which enables anomalous reflections together with the high-efficiency frequency conversion. In this specific application, an STC digital metasurface composed of *N* columns of programmable elements is considered for a 2D scenario (see Figure 3(a)); the reflection phase of each element is modulated by switching the embedded PIN diodes. The STC digital metasurface is illuminated by an obliquely incident plane wave with the time-harmonic dependence exp(*j*2*πf*_c_*t*_c_), transverse magnetic polarization, and incident angle *θ*_i_. The reflection coefficient of the *p*^th^ element is temporally modulated, which can be expressed as a periodic function Γ_*p*_(*t*) = ∑_*n*=1,⋯,*L*_Γ_*p*_^*n*^*U*_*p*_^*n*^(*t*), with *U*_*p*_^*n*^(*t*) representing a rectangular pulse function with a modulation period *T*_0_. Hence, the phase response of each element can be characterized by a set of time-coding sequences with length *L*, and the whole metasurface is further represented by an STC matrix. According to the previously introduced theory of STC digital metasurfaces in Section 2, the time-domain far-field scattering pattern in this circumstance can be approximately written as

(6) $$\begin{array}{c} f\left(\theta ,t\right)=E\left(\theta \right)\overset{N}{\underset{p=1}{\sum }}\Gamma_{p}\left(t\right)\exp\left[j\frac{2\pi }{\lambda_{\text{c}}}\left(p-1\right)d\left(\sin\theta +\sin\theta_{\text{i}}\right)\right], \end{array}$$

where *E*(*θ*) = cos*θ* is the far-field scattering pattern (approximated by a cosine function) of the *p*^th^ programmable element at the center frequency, *d* denotes the element spacing, and other parameters are the same as those defined in equation (1). By taking the Fourier series expansion of the periodic function Γ_*p*_(*t*), the frequency-domain far-field patterns of the STC digital metasurface at the harmonic frequency *f*_c_ + *mf*_0_ can be expressed as

(7) $$\begin{array}{c} F_{m}\left(\theta \right)=E\left(\theta \right)\overset{N}{\underset{p=1}{\sum }}a_{p}^{m}\exp\left[j2\pi \left(p-1\right)d\left(\frac{\sin\theta }{\lambda_{\text{r}}}+\frac{\sin\theta_{\text{i}}}{\lambda_{\text{c}}}\right)\right], \end{array}$$

where *λ*_r_ = *c*/(*f*_c_ + m*f*_0_) denotes the wavelength of the *m*^th^ harmonic wave, and *a*_*p*_^*m*^ represents the Fourier coefficients of Γ_*p*_(*t*):

(8) $$\begin{array}{c} a_{p}^{m}=\overset{L}{\underset{n=1}{\sum }}\frac{\Gamma_{p}^{n}}{L}\mathrm{sinc}\left(\frac{\pi m}{L}\right)\exp\left[\frac{-j\pi m\left(2n-1\right)}{L}\right]. \end{array}$$

**Figure 4:**
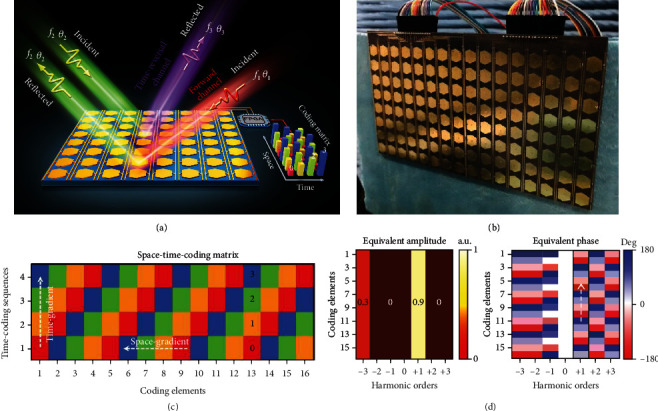
Breaking reciprocity via STC digital metasurfaces. Reproduced from [113]. (a) Conceptual illustration of realizing nonreciprocal reflection effect based on an STC digital metasurface. (b) Photograph of the fabricated 2-bit programmable metasurface. (c) 2-bit STC matrix with dimension 16 × 4. (d) Equivalent amplitudes and phases for the STC matrix in (c).

Figure 4(a) schematically illustrates the process of nonreciprocal effects in a two-port reflection system based on the STC digital metasurface. By judiciously engineering the spatiotemporally gradient coding sequence, a plane wave with frequency *f*_1_ obliquely incident from the angle *θ*_1_ will be reflected at the angle *θ*_2_ and frequency *f*_2_. While for the time-reversal case, a plane wave with frequency *f*_2_ incident from the angle *θ*_2_ will be reflected at the angle *θ*_3_ and frequency *f*_3_. Due to the spatiotemporal modulation, the angle *θ*_3_ and frequency *f*_3_ are different from the original angle *θ*_1_ and frequency *f*_1_ (i.e., *θ*_3_ ≠ *θ*_1_, *f*_3_ ≠ *f*_1_), which obviously breaks the Lorentz reciprocity [113].

To this end, we further assume that the STC programmable metasurface encompasses 16 columns of elements, which have 2-bit time-coding sequences with the length *L* = 4. Figure 4(b) displays a microwave prototype of a 2-bit STC digital metasurface. Figure 4(c) shows the 2-bit STC matrix, in which each coding element is controlled by a time gradient phase with an interval of *π*/2 and the time-coding sequences across the metasurface are progressively shifted by a time interval *T*_0_/4 so that every two adjacent elements always exhibit a *π*/2 phase difference in each time interval. The corresponding equivalent amplitudes and phases (calculated via equation (8)) are shown in Figure 4(d), from which we can observe that this spatiotemporal gradient coding essentially leads to an anomalous reflection with incident EM energy mainly concentrated at the positive first harmonic (*m* = 1). Accordingly, the effective phase gradient at the +1^st^ harmonic frequency can be written as

(9) $$\begin{array}{c} \frac{\partial \psi }{\partial x}=-\frac{\pi }{2d}. \end{array}$$

Recalling the forward scenario in Figure 4(a), for a plane wave with frequency *f*_c_ obliquely incident from an angle *θ*_1_, the dominant reflected power will be at the +1^st^ harmonic frequency *f*_c_ + *f*_0_ and at an angle *θ*_2_, given by

(10) $$\begin{array}{c} \theta_{2}=\arcsin\left(\frac{\sin\theta_{1}-\lambda_{\text{c}}/4d}{1+f_{0}/f_{\text{c}}}\right). \end{array}$$

When considering the time-reversal scenario in Figure 4(a), for a plane wave with frequency *f*_c_ + *f*_0_ incident from the angle *θ*_2_, the dominated reflected waves will not follow the original incident direction in the forward channel but mainly exist at a different frequency *f*_c_ + 2*f*_0_ and an angle *θ*_3_, given by

(11) $$\begin{array}{c} \theta_{3}=\arcsin\left(\frac{\sin\theta_{1}}{1+2f_{0}/f_{\text{c}}}\right). \end{array}$$

The reflected wave in the time-reversal case no longer propagates along the direction of the original incident wave in the forward case and also brings out a frequency shift of 2*f*_0_. That is to say, for the oblique incidence (i.e., *θ*_1_ ≠ 0), the time-reversal reflection channel and the forward incident channel are isolated in both the spectral and spatial domains. It can be observed from equation (10) that the angular difference |*θ*_3_ − *θ*_1_| increases with the frequency ratio *f*_0_/*f*_c_ and the original incident angle *θ*_1_.

**Figure 5:**
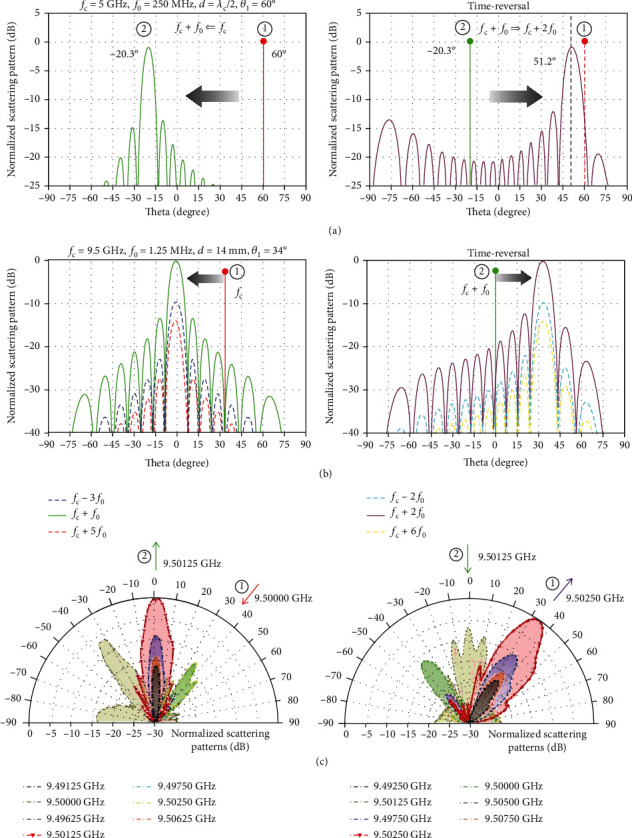
Programmable nonreciprocal effect and frequency conversion. Reproduced from [113]. (a) Numerically calculated scattering patterns at various frequencies for the forward reflection scenario (excited from Port 1 at *f*_c_ and *θ*_i_ = 60°) and time-reversal reflection scenario (excited from Port 2 at *f*_c_ + *f*_0_ and *θ*_i_ = 20.3°), respectively. (b) Numerically calculated scattering patterns at various frequencies for the forward reflection scenario (excited from Port 1 at *f*_c_ and *θ*_i_ = 34°) and time-reversal reflection scenario (excited from Port 2 at *f*_c_ + *f*_0_ and *θ*_i_ = 0°), respectively. (c) Measured scattering patterns at various frequencies for the forward and time-reversal reflection scenarios, respectively.

Here, we consider the first representative example of the nonreciprocal reflection by assuming that *f*_c_ = 5 GHz, *f*_0_ = 250 MHz, *d* = *λ*_c_/2, and *θ*_1_ = 60°. In this circumstance, the reflected angles in the forward and time-reversal scenarios are theoretically calculated (via equations (10) and (11)) as *θ*_2_ = 20.40° and *θ*_3_ = 51.93°, respectively.  Figure 5(a) shows the numerically computed scattering patterns (via equation (7)) in the forward and time-reversal cases. It can be clearly seen that angular separation between *θ*_3_ and *θ*_1_ reaches a sizable value of ∼9°. For this parameter configuration, the nonreciprocal effect can separate the wave reflections spatially and spectrally. However, the modulation frequency *f*_0_ = 250 MHz of the time-coding sequence in Figure 4(c) corresponds to the switching rate 4*f*_0_ = 1 GHz of PIN diodes, which is not currently available with commercial PIN diodes.

Furthermore, we consider a practical example with the more feasible parameters of *f*_c_ = 9.5 GHz, *f*_0_ = 1.25 MHz, *d* = 14 mm, and *θ*_1_ = 34°.  Figure 5(b) displays the corresponding scattering patterns in the forward and time-reversal cases, for which the angular difference between *θ*_3_ and *θ*_1_ is <1°. Although this very small angular difference could not be distinguished in space with the current experimental setup, it is hopeful to experimentally observe the frequency isolation in the spectral domain by a spectrum analyzer with high precision. To experimentally verify this example, a prototype of a 2-bit STC digital metasurface working around the frequency of 9.5 GHz was manufactured (see [113] for details), as displayed in Figure 4(b).  Figure 5(c) shows the measured scattering patterns in the forward and time-reversal cases, from which the reflected angles and harmonic distributions show great agreement with the theoretical results, thereby providing the first experimental verification of the nonreciprocal effect enabled by spatiotemporally modulated metasurfaces.

It is worth highlighting that the FPGA-controlled STC digital metasurface lays the foundation for the “on-demand” realization of the nonreciprocal effect and frequency conversion in a completely programmable fashion, which may find many promising applications to the mixer, duplexer, optical isolator, unidirectional transmission, Doppler illusion, wireless communication, and radar systems.

### 3.4. Generation of Arbitrary Multibit Programmable Phases

As aforementioned in Section 3.1, multibit programmable metasurfaces exhibit low phase quantization error and can obtain more precise control of EM waves and thus have a greater capability to realize some sophisticated EM functions. Up to now, programmable metasurfaces with 1-bit and 2-bit phases have been effectively engineered by integrating with active devices and FPGA. However, it is still difficult to realize multibit (i.e., 3-bit or higher-bit) programmable metasurfaces via PIN diodes. Some alternative techniques have also been exploited for programmable metasurfaces, such as varactors, liquid crystal, graphene, and microelectromechanical systems (MEMS), but all those strategies have limited phase coverages and do not allow the continuous 2*π* phase modulation.

**Figure 6:**
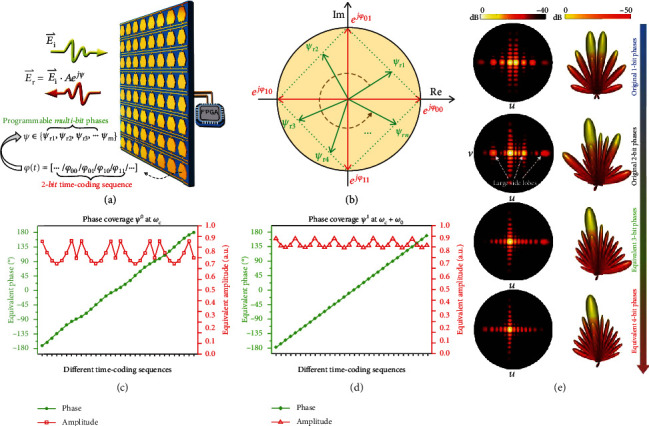
Generation of arbitrary multibit programmable phases. Reproduced from [114]. (a) Conceptual illustration of arbitrary multibit programmable metasurfaces under the control of an FPGA. (b) Vector synthesis in the complex plane for generating the multibit programmable phases. Equivalent phase coverage: (c) at the central frequency and (d) at the +1^st^ harmonic frequency. (e) Comparison of 3D scattering patterns with respect to the original 1-bit and 2-bit phases and equivalent 3-bit and 4-bit phases for beam steering.

In a recent study [114], a physical programmable metasurface using the 2-bit time-coding strategy was put forward to achieve arbitrary multibit and nearly quasicontinuous phases, as illustrated in Figure 6(a). Figure 6(b) shows the schematic of the vector synthesis method, which was originally proposed to analyze the phase coverages. With the aid of this method, 4-bit and arbitrary multibit phases can be synthesized through a physical 2-bit programmable metasurface. The reflection coefficients (i.e., *e*^*jφ*_00_^, *e*^*jφ*_01_^, *e*^*jφ*_10_^, and *e*^*jφ*_11_^) of the original 2-bit case are represented by four basic vectors (red arrows) in a complex plane, as shown in Figure 6(b). By carefully selecting the time-coding sequences, the new vectors (green arrows in Figure 6(b)) with arbitrary phases *ψ*_r*n*_ can be successfully synthesized by suitably combining the basic vectors [114]. Figures 6(c) and 6(d) indicate that the 2*π* phase coverage can be realized with high amplitudes at both the central and +1^st^ harmonic frequencies just by properly designing 2-bit time-coding sequences. Moreover, some examples of beam steering are numerically investigated to verify the effectiveness of the time-coding strategy. Figure 6(e) shows the far-field scattering patterns of beam steering with respect to the original 1-bit and 2-bit phases and equivalent 3-bit and 4-bit phases, from which the significant quantization lobes of the original 1-bit and 2-bit phases are noticeable, but the side lobes of the equivalent 4-bit phases are well avoided. For experimental verification, an FPGA-controlled microwave prototype was manufactured and characterized, and the measured results show good agreement with the numerical analysis.

Overall, the time-coding strategy provides a new path to design the arbitrary multibit programmable metasurfaces, without the need for complicated physical design and control circuit systems, which will find potential applications in high-resolution imaging, high-performance antennas, and high-capacity communications and radars. This strategy can also be extended to other frequency regimes (such as terahertz, optics, and acoustics) and generalized to obtain multibit programmable transmission phases.

### 3.5. Nonlinear Harmonic Manipulations via TDC Digital Metasurfaces

**Figure 7:**
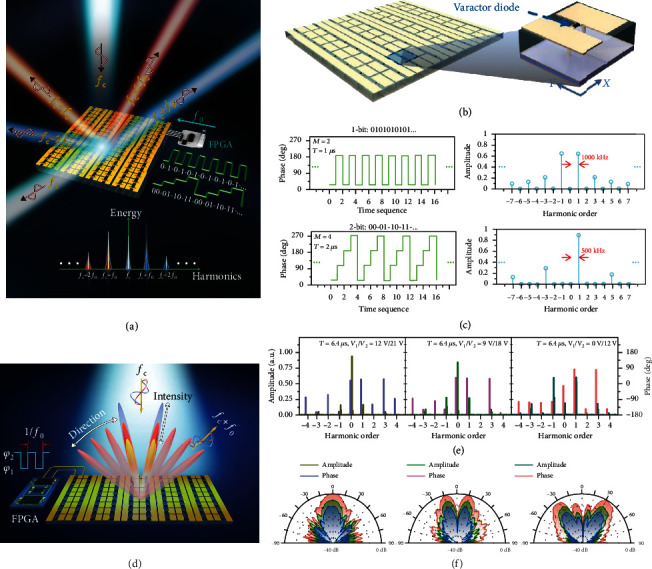
Nonlinear harmonic manipulations via TDC digital metasurfaces. (a) Conceptual illustration of TDC digital metasurfaces for spectral manipulations. Reproduced from [109]. (b) Geometry of the varactor-based programmable metasurface and its element. Reproduced from [109]. (c) The calculated spectral intensities pertaining to phase modulation waveforms with 1-bit (“010101…”) and 2-bit (“00-01-10-11…”) time-coding sequences. Reproduced from [109]. (d) Conceptual illustration of TDC digital metasurfaces for independent manipulation of harmonic phases and amplitudes. Reproduced from [120]. (e) Measured spectral phases and amplitudes at various harmonics, corresponding to three different voltage combinations *V*_1_/*V*_2_. Reproduced from [120]. (f) Measured scattering patterns at the +1^st^ harmonic frequency, corresponding to three sets of spatial coding “00000000,” “00001111,” and “00110011,” respectively. Reproduced from [120].

In addition to using PIN diodes, time-varying metastructures can also be implemented via the use of varactors, which provides more control states in the temporal modulation. As a key branch of STC digital metasurfaces, the TDC digital metasurface was put forward to efficiently manipulate the nonlinear harmonic distributions of EM waves [109, 120], as illustrated in Figure 7(a). In such a study [109], only time-coding is considered, in which case the space-invariant reflection coefficient of the whole metasurface is temporally modulated in accordance with the same function Γ(*t*) with a period *T*_0_ = 1/*f*_0_. By assuming that the TDC digital metasurface is normally illuminated by a monochromatic incident plane wave with the time-harmonic form of exp(*j*2*πf*_c_*t*_c_), the basic theory in Section 2 can be generalized to represent the reflected wave *E*_r_(*f*) in the frequency domain as follows:

(12) $$\begin{array}{c} E_{\text{r}}\left(f\right)=E_{\text{i}}\left(f\right)*\Gamma \left(f\right)=\delta \left(f-f_{\text{c}}\right)*\overset{\infty }{\underset{k=-\infty }{\sum }}a_{k}\delta \left(f-{kf}_{0}\right)=\overset{\infty }{\underset{k=-\infty }{\sum }}a_{k}\delta \left(f-f_{\text{c}}-{kf}_{0}\right), \end{array}$$

where ∗ is the symbol of convolution operation, *E*_i_(*f*) denotes the incident wave, *δ*(*f*) denotes the Dirac delta function, and *a*_*k*_ represents the Fourier coefficient of the periodic function Γ(*t*) at the *k*^th^-order harmonic. It can be observed from equation (12) that the reflected spectrum *E*_r_(*f*) is a linear superposition of the EM fields at many harmonic frequencies *f*_c_ + *kf*_0_.

Generally, the TDC digital metasurface can be used to generate arbitrarily nonlinear harmonic distributions by elaborately customizing the time-modulated waveforms [109], which opens up a new path to realize the EM spectral shaping. Figure 7(b) shows the basic geometry of the TDC digital metasurface, in which each coding element consists of two rectangular patches connected by a varactor. Some representative examples obtained by a varactor-based TDC digital metasurface were presented to illustrate the working principles. Figure 7(c) displays the spectral intensity distributions regarding the phase modulation waveforms with 1-bit (“010101…”) and 2-bit (“00-01-10-11…”) time-coding sequences. For the 1-bit time-coding sequence “010101…,” the incident power is only transferred to the odd harmonics with the symmetric spectral distribution. But for the 2-bit time-coding sequence “00-01-10-11…,” most of the incident energy is spread to the +1^st^ harmonic with the asymmetric spectral distribution. More details on amplitude modulation can be found in [109]. Overall, both the numerical and experimental results demonstrated the capability of the TDC digital metasurfaces in manipulating the EM spectral distribution, which will find interesting applications in the Doppler shift, velocity illusion, and simplified architecture wireless communication systems.

As can be understood from the complex-valued equivalent coefficients *a*_*pq*_^*m*^ in equation (4), one major limitation of STC digital metasurfaces originates from the *inherent strong coupling* between the phases and amplitudes of various harmonics. Hence, a recent study on TDC digital metasurfaces was proposed to regulate the amplitudes and phases at different frequencies independently via different time-varying signals [120].  Figure 7(d) shows the conceptual illustration of a reflective TDC digital metasurface, whose reflection phase is periodically modulated in the time domain with a square wave varying between two predesigned phase values *ϕ*_1_ and *ϕ*_2_ (corresponding to the biasing voltages *V*_1_ and *V*_2_). To overcome the coupling between the harmonic amplitudes and phases, a time delay *t*_0_ is brought in the time-domain reflection coefficient Γ(*t*), leading to an additional phase shift −2*πkf*_0_*t*_0_ with unchanged amplitudes at the *k*^th^ harmonic frequency [110, 120]. After some mathematical derivation, the spectral distribution of reflected waves can be expressed as

(13) $$\begin{array}{c} E_{\text{r}}\left(f\right)=2\pi A\cos\left(\frac{\phi_{1}-\phi_{2}}{2}\right)\exp\left[j\left(\frac{\phi_{1}+\phi_{2}}{2}\right)\right]E_{\text{i}}\left(f\right)+\overset{\infty }{\underset{h=-\infty }{\sum }}\left(\frac{4A}{2h-1}\right)\sin\left(\frac{\phi_{1}-\phi_{2}}{2}\right)\exp\left\{j\left[\frac{\phi_{1}+\phi_{2}}{2}-\left(2h-1\right)2\pi {\text{ft}}_{0}\right]\right\}E_{\text{i}}\left[f-\left(2h-1\right)f_{0}\right], \end{array}$$

where *A* is a constant value of reflection amplitudes and *h* is an integer. It can be observed from equation (13) that the spectral components of reflected waves only exist at the central frequency (*k* = 0) and odd-harmonic frequencies (*k* = ±1, ±3, ±5, ⋯) due to the Fourier transform characteristic of square waves [120]. From equation (13), we can notice that the amplitudes of reflected waves at the central and odd-harmonic frequencies can be controlled by tailoring the phase difference (*ϕ*_1_ − *ϕ*_2_). Besides, the time delay *t*_0_ provides additional degrees of freedom to control the phases at the odd-harmonic frequencies, which keeps the amplitude unchanged and thereby enables independent manipulations of harmonic phases and amplitudes.

For experimental verification, a microwave prototype of varactor-based TDC digital metasurfaces controlled by FPGA was fabricated and characterized in [120]. Figure 7(e) displays the measured spectral phases and amplitudes at various harmonics, corresponding to three different voltage combinations *V*_1_/*V*_2_ (applied to the entire metasurface). The amplitude of the +1^st^ harmonic is associated with the biasing voltage combinations, which can obtain a dynamic range of 25 dB by tuning the external biasing voltages [120]. Next, different time delays 0 and *T*_0_/2 are assigned to different columns of the metasurface elements for spatial coding “0” and “1,” which can be used for beam shaping. Figure 7(f) shows three groups of measured scattering patterns at the +1^st^ harmonic frequency pertaining to three sets of spatial coding “00000000,” “00001111,” and “00110011,” respectively. It can be obviously observed that the power intensities of scattering patterns are controlled by the voltage combinations *V*_1_/*V*_2_, while the beam shapes remain unchanged. The proposed approach has potential applications in wireless communication; more details will be discussed in Section 4.

### 3.6. Frequency Synthesis, Polarization Conversion, and Convolution Operation

**Figure 8:**
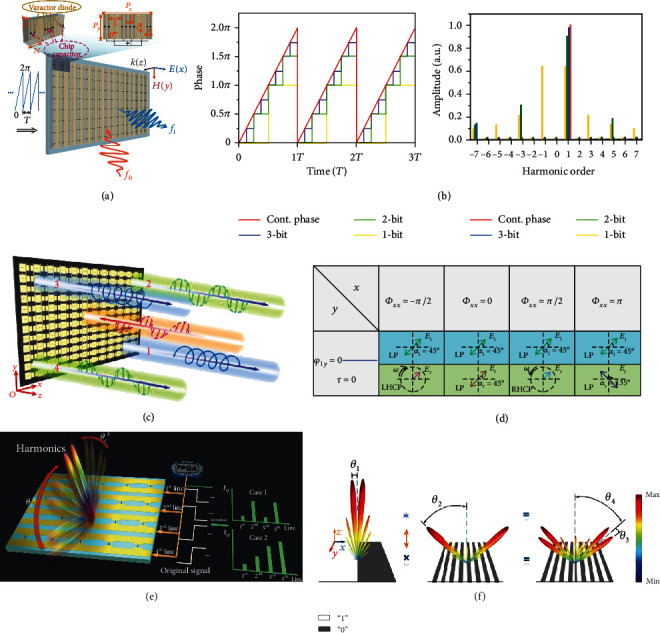
Frequency synthesis, polarization conversion, and convolution operation. (a) Conceptual illustration of STC digital metasurfaces for frequency synthesis. Reproduced from [121]. (b) The time-modulated waveform with 1-bit, 2-bit, and 3-bit quantized phases and continuous sawtooth wave phases, as well as the corresponding harmonic amplitude distributions. Reproduced from [109]. (c) Conceptual illustration of TDC digital metasurfaces for polarization conversion. Reproduced from [122]. (d) A polarization conversion diagram with some specific collocations of the *x*- and *y*-polarized phases, from which the RHCP, LHCP, and cross-LP are successfully synthesized. Reproduced from [122]. (e) Conceptual illustration of TDC digital metasurfaces for convolution operation. Reproduced from [123]. (f) An illustrative example of nonlinear convolution operation at the +1^st^ harmonic frequency, as it can be observed that the original dual-beam pattern deviates from the normal axis to the angle *θ*_2_ with negligible distortion. Reproduced from [123].

Some other interesting applications of the STC digital metasurfaces include frequency synthesis, polarization conversion, and convolution operation. In [121], an STC digital metasurface integrated with varactors and chip capacitors was proposed to synthesize the ±1^st^ harmonic frequencies of reflected waves with high efficiency. As conceptually illustrated in Figure 8(a), the reflection phase of the entire metasurface is periodically modulated with a sawtooth waveform in the temporal domain. In some application scenarios, unwanted high-order harmonics need to be suppressed to avoid spectrum pollution. Figure 8(b) shows the time-modulated waveform with 1-bit, 2-bit, and 3-bit quantized phases and continuous sawtooth wave phases, as well as the corresponding harmonic amplitude distributions. We notice that the amplitude of the +1^st^ harmonic gradually increases as the time-modulated waveform changes from 1-bit discrete phases to continuous phases during one period. In other words, higher quantization bits can be employed to suppress the undesired harmonic components. Actually, the 3-bit quantized phase can be used as a compromise solution for harmonic conversion with an efficiency of ~95%. Besides, this high-efficiency harmonic conversion combined with the approach of time delay can also be exploited for some beam shaping applications. The measured results presented in [121] agree well with the theoretical analyses, which indicates that the conversion efficiency can reach ~88% and the harmonic suppression ratio is ~21 dB. This spatial frequency synthesizer based on the STC digital metasurface may find important applications in future wireless communication systems. More recently, some similar designs about frequency translation enabled by time-modulated metasurfaces have also been presented in [54–56].

The aforementioned STC and TDC digital metasurfaces can only work in the scenarios of single polarization. In Ref. [122], an anisotropic TDC digital metasurface working at dual polarizations was proposed to realize the programmable polarization conversions at both the fundamental and harmonic frequencies. By applying the theory of nonlinear harmonics introduced in Section 3.5, a general theory of polarization regulation was proposed to synthesize the linear and nonlinear polarization conversions, including the arbitrarily linearly polarized (LP), cross-LP, right-handed circularly polarized (RHCP), and left-handed circularly polarized (LHCP) reflected waves. Due to the dual-polarized structural design of programmable elements (see Figure 4(a) in [122]), the time-varying square wave reflection coefficients Γ_*xx*_(*t*) and Γ_*yy*_(*t*) along two orthogonal directions (i.e., the *x*- and *y*-polarized directions) of the metasurface can be independently controlled. As conceptually illustrated in Figure 8(c), the anisotropic TDC digital metasurface is normally illuminated by an incident wave with arbitrary linear polarizations (represented by the orange ray). By adjusting the phases of Γ_*xx*_(*t*) and Γ_*yy*_(*t*), RHCP, arbitrarily LP, LHCP, and cross-LP reflected waves can be generated, as, respectively, represented by blue ray 1, green ray 2, blue ray 3, and green ray 4. Figure 8(d) shows a polarization conversion diagram with some specific collocations of different *x*- and *y*-polarized phases, from which RHCP, LHCP, and cross-LP are successfully synthesized. More theoretical results can be found in [122]. For experimental validation, a microwave prototype of the anisotropic TDC digital metasurface was manufactured. The measured results are in great agreement with the theoretical analysis, verifying the feasibility of the proposed polarization manipulation scheme.

A convolution theorem was first introduced in SDC digital metasurfaces for realizing flexible scattering pattern shifts [90], which can steer a beam pattern to an arbitrary spatial direction. In a recent study [123], a time-domain convolution theorem based on a TDC digital metasurface was proposed to achieve the scattering pattern shift at the harmonic frequencies, as illustrated in Figure 8(e). As previously mentioned, a time delay introduced in the time-domain reflection coefficient can result in an additional phase shift −2*πkf*_0_*t*_0_ at the *k*^th^ harmonic frequency [110, 120]. Figure 8(f) illustrates an example of nonlinear convolution operation at the +1^st^ harmonic frequency, as it can be observed that the original dual-beam pattern deviates from the normal axis to the angle *θ*_2_ with negligible distortion. The working process is the same as that in [90], and more results can be found in [123].

### 3.7. Harmonic Information Transitions and Independent Control of Dual Harmonics

**Figure 9:**
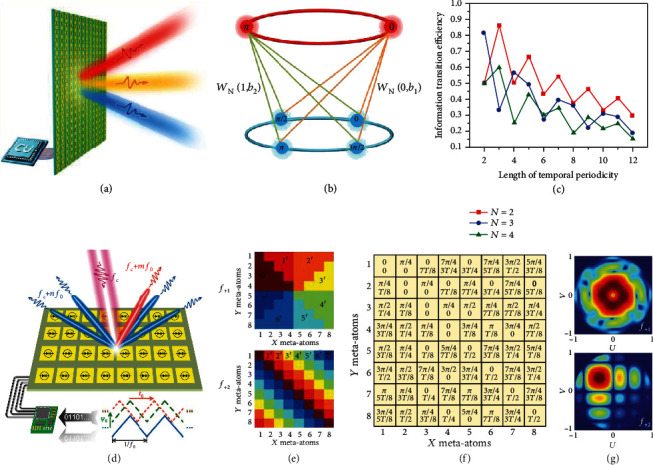
Harmonic information transitions and independent control of dual-harmonic waves. (a) Conceptual illustration of manipulating wave transmission via an STC digital metasurface. Reproduced from [124]. (b) Schematic of independent control of phase states at two frequencies. Reproduced from [124]. (c) Numerically calculated information transition efficiency, pertaining to different lengths of the time-coding sequences and the number of input phase states. Reproduced from [124]. (d) Conceptual illustration of dual-harmonic manipulation based on an STC digital metasurface. Reproduced from [125]. (e) A spiral-like phase distribution at the +1^st^ harmonic frequency and a diagonal gradient distribution at the +2^nd^ harmonic frequency, respectively. Reproduced from [125]. (f) The corresponding combinations of initial phases and time delays. Reproduced from [125]. (g) The corresponding numerically calculated 2D scattering patterns at the +1^st^ and +2^nd^ harmonic frequency, respectively. Reproduced from [125].

From the perspective of information technology, the STC digital metasurface can be regarded as an information processer, which can transform the spatiotemporally modulated signals to spatial-spectral information at various harmonics. In a recent study [124], the information transition mechanisms of STC digital metasurfaces were proposed, in which the idea of group extension and independent control of dual-harmonic waves are characterized as two major tools to analyze the harmonic information transitions.  Figure 9(a) shows the conceptual illustration of manipulating wave transmission via an STC digital metasurface. Referring to [124] for details, the group extension mechanism is used to expand the phase states of coding elements via the factor *q*, which can realize more precise control of EM waves without the need for complicated metasurface design. To some extent, this idea is essentially similar to the concept of multibit programmable phases [114], as previously presented in Section 3.4. This analytical method of group theory provides a new perspective to understand equation (4) proposed in [110].

Besides, the independent control of the spectral responses of the STC digital metasurface is also demonstrated.  Figure 9(b) shows the schematic of independent control of phase states at two frequencies, in which the same colored lines connect the phase states from the central frequency to the +1^st^ harmonic frequency. By using the permutation and translation operations in the same time-coding sequence [124], the phases of the output spectral responses can be independently controlled at two frequencies. Actually, the proposed permutation and translation, respectively, work the same as the initial phase and time delay introduced in the time-coding sequences [125], which will be discussed later in this section. Moreover, the information transition efficiencies of the STC digital metasurfaces regarding the aforementioned two mechanisms were also investigated by incorporating Shannon's entropy theory [124].  Figure 9(c) shows the numerically calculated information transition efficiency, pertaining to different lengths of the time-coding sequences and the number of input phase states. The theoretical results can be applied to predict the channel capacity of the STC digital metasurfaces, which offers potential guidance for future wireless communications.

In Ref. [125], an STC strategy with controllable initial phases and time delays was proposed to simultaneously control dual harmonics, leading to independent beam control at arbitrary two harmonics.  Figure 9(d) shows a conceptual illustration of dual-harmonic manipulation based on an STC digital metasurface. By changing the initial phase *ψ*_0_ and time delay *t*_0_ in the time-modulated phase periodic function, one can independently manipulate beam shaping at two harmonic frequencies. Referring to [125] for more details, the theoretical derivations show that the initial phase *ψ*_0_ has the same effect on the phases of all harmonics but the time delay *t*_0_ induces different phase shifts with respect to harmonic orders, and neither of them has any effect on the amplitudes of harmonics. As an illustrative example, Figure 9(e) shows a spiral-like phase distribution at the +1^st^ harmonic frequency and a diagonal gradient distribution at the +2^nd^ harmonic frequency.  Figure 9(f) shows the corresponding combinations of initial phases and time delays for this example. The phases of STC digital metasurfaces are temporally modulated by parameters in Figure 9(f), which generates a vortex beam at the +1^st^ harmonic frequency and a single beam with a deflection angle at the +2^nd^ harmonic frequency, as shown in Figure 9(g). These numerical examples were also validated by experiments, which demonstrated the effectiveness of dual-harmonic control, leading to potential applications in multiuser wireless communications.

### 3.8. Joint Multifrequency Beam Shaping and Steering

As previously discussed in Section 3.1, harmonic beam steering at multiple frequencies can be obtained by using brute-force numerical optimization (e.g., BPSO algorithm). However, the applicability of optimization methods is strictly limited by the computational complexity and nonlinear feature. One limitation of conventional STC schemes is the inherent entanglement of multifrequency synthesis [110], which originates from equation (4) that the equivalent phases and amplitudes at each harmonic frequency are influenced by all the reflection coefficients in the time-coding sequences. Therefore, the basic STC schemes cannot attain the independent and simultaneous syntheses of the prescribed beam patterns at multiple frequencies. To overcome this limitation, a new coding strategy was put forward to achieve joint multifrequency beam control [126]. This strategy depends on the sophisticated time-coding sequences with properly designed and temporally intertwined subsequences, which can efficaciously disentangle the joint multifrequency syntheses.

**Figure 10:**
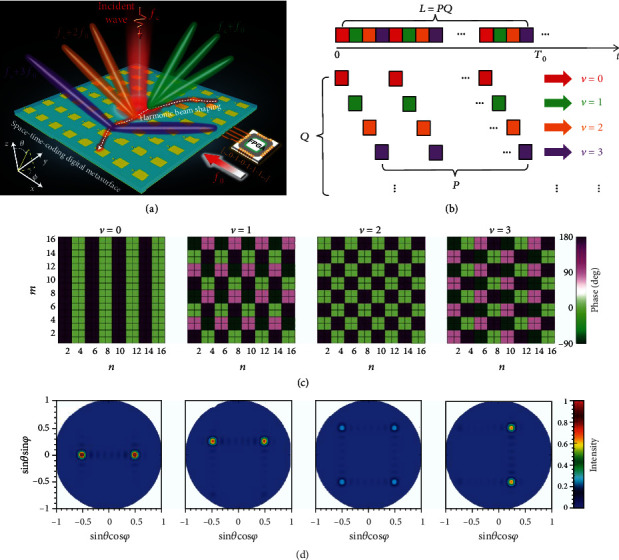
Joint multifrequency beam shaping and steering. Reproduced from [126]. (a) Conceptual illustration of multifrequency joint syntheses via an STC digital metasurface. (b) Schematics of intertwined coding subsequences. As denoted by the color coding, each subsequence only has an effect on one specific harmonic. (c) The synthesized phase patterns for the complex-valued equivalent amplitude *a*_*ν*_^(*nm*)^ at the first four frequencies. (d) The corresponding numerically calculated 2D scattering patterns.

As illustrated in Figure 10(a), an STC digital metasurface combined with the new coding strategy can synthesize independently and simultaneously the desired scattering patterns (e.g., different beam numbers, directions, and shapes) at desired multiple frequencies. In [126], the reflection phases of the STC digital metasurface are quantized by the number *S* over the interval (0, 360°). Thus, for a 1-bit case (*S* = 2), we can obtain Γ_*l*_^(*nm*)^ ∈ {1, −1} corresponding to the digits {0, 1}, while for a 2-bit case (*S* = 4), we can obtain Γ_*l*_^(*nm*)^ ∈ {1, *j*, −1, −*j*} corresponding to the digits {0, 1, 2, 3}, and so forth. Figure 10(b) schematically illustrates the proposed strategy of intertwined coding subsequences. As assumed in [126], the problem is formulated as the joint *Q*-harmonic syntheses (i.e., orders *ν* = 0, 1, ⋯, *Q* − 1). To this end, each time-coding sequence is decomposed into *Q* sets of intertwined coding subsequences with the length *P*; thus, the total length of the time-coding sequence is *L* = *PQ* in one period *T*_0_. The time-coding sequence composed of intertwined subsequences can be expressed as

(14) $$\begin{array}{c} \Gamma_{q+1+pQ}=\Omega_{pq}\Gamma_{q+1},\;p=0,\cdots ,P-1,\;q=0,\cdots ,Q-1, \end{array}$$

where *Ω*_*pq*_ are phase shift factors with the same quantization as the original reflection states Γ_*q*+1_. Thus, the equivalent complex-valued amplitudes in equation (4) can be rewritten as

(15) $$\begin{array}{c} a_{\nu }=c_{\nu }\overset{Q-1}{\underset{q=0}{\sum }}\Gamma_{q+1}\alpha_{q\nu }\exp\left(-\frac{j2\pi \nu q}{L}\right), \end{array}$$

where *c*_*ν*_ = 1/*L* · sinc(*πν*/*L*)exp(−*jπν*/*L*) governs the decay of the spectrum, and *α*_*qν*_ are regarded as digital filter terms and can be expressed as

(16) $$\begin{array}{c} \alpha_{q\nu }=\overset{P-1}{\underset{p=0}{\sum }}\Omega_{pq}\exp\left(-\frac{j2\pi \nu p}{P}\right). \end{array}$$

In order to disentangle the multifrequency syntheses, *α*_*qν*_ need to be designed by judiciously selecting the phase shift factors *Ω*_*pq*_. Ideally, this would require $\alpha_{q\nu }\propto {\tilde{\delta }}_{q\nu }$ with ${\tilde{\delta }}_{q\nu }$ denoting a reduced Kronecker delta. In this case, each coding subsequence (represented by the same colored square in Figure 10(b)) only basically affects one specific harmonic, thereby realizing joint syntheses at multiple frequencies independently and simultaneously. Referring to [126] for details, this synthesis problem has a very simple solution when the number *Q* of harmonic frequencies of interest is not greater than the number *S* of quantized reflection phases, so we should choose *P* = *S* and *Ω*_*pq*_ = exp(*j*2*πpq*/*S*) in this case.

To show the potential of this new coding strategy, in [126], we consider a 2-bit (*S* = 4) STC digital metasurface with 16 × 16 programmable elements. For an exact closed-form solution, we apply the proposed approach for joint syntheses at *Q* = 4 harmonic frequencies (i.e., *ν* = 0, 1, 2, 3) and assume that the length of each intertwined subsequence is *P* = 4; thereby, the total length of the time-coding sequence is *L* = *PQ* = 16 in one period. As an illustrative example, Figure 10(c) shows the synthesized phase patterns for the equivalent complex-valued amplitude *a*_*ν*_^(*nm*)^ at the first four harmonic frequencies, whereas Figure 10(d) displays the corresponding 2D scattering patterns. It can be clearly observed that different scattering patterns at the four frequencies of interest are independently synthesized; more illustrative examples about diffuse and OAM-type scattering patterns can be found in [126]. For the experimental proof of concept, the previous prototype of a 2-bit STC digital metasurface in [113] was utilized again to validate the feasibility of the joint multifrequency syntheses. The measured patterns at different frequencies were in good agreement with the numerical simulations.

Overall, the proposed approach of joint multifrequency syntheses remarkably enhances the wavefront manipulation capabilities of STC digital metasurfaces, which paves the way for promising applications in wireless communication, radar, imaging, and some more advanced information systems. It is worth emphasizing that the proposed coding strategy inherits the character of programmability in the digital metasurface platform, and thus, it is expected to dynamically alter the synthesized scattering patterns or redistribute the harmonic spectrum “on demand,” by changing the coding sequences preloaded in an FPGA.

### 3.9. New Architecture Wireless Communication Systems

One of the most exciting and intriguing applications of STC digital metasurfaces is the construction of new architecture wireless communication systems. As a branch of STC digital metasurfaces, the TDC digital metasurfaces only consider the temporal coding, in which no spatial coding is assumed. The above analyses in Section 3.5 show that TDC digital metasurfaces have powerful capabilities in accurately engineering the harmonic amplitudes and phases of the reflected EM waves. The TDC digital metasurface can process the digital information at the interface, in which the baseband signal can be modulated on the carrier wave directly without the need for digital-analog conversion and mixing processes. This direct modulation based on metasurfaces can be realized by establishing a mapping relation between the baseband signals and the time-varying reflection coefficients.

**Figure 11:**
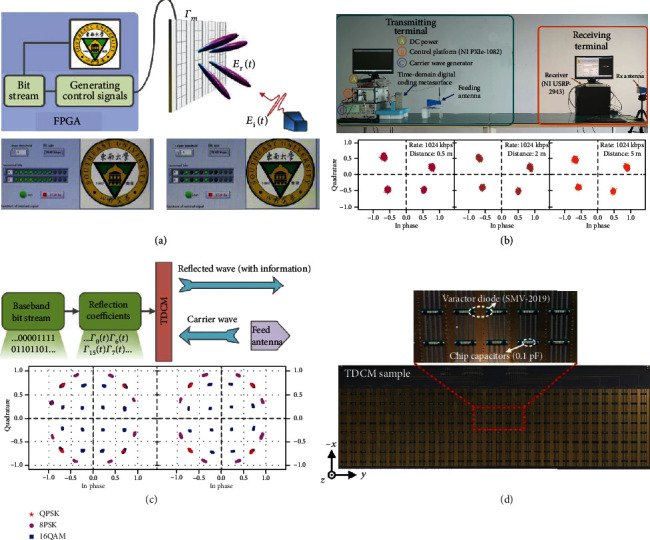
New architecture wireless communication systems via TDC digital metasurfaces. (a) Schematic of the metasurface-based transmitter of the BFSK wireless communication system and the corresponding measured results. Reproduced from [109]. (b) Photo of the wireless communication system and the measured constellation diagrams. Reproduced from [95]. (c) The block diagram of the communication transmitter constructed by a TDC digital metasurface and the constellation diagram of QPSK, 8PSK, and 16QAM. Reproduced from [96]. (d) The fabricated microwave prototype of the TDC digital metasurface working around the frequency of 4.25 GHz. Reproduced from [96].

For instance, a new architecture wireless communication system with a binary frequency shift keying (BFSK) modulation scheme was proposed in [109]. The TDC digital metasurface can transfer the power from the carrier frequency to the ±1^st^ harmonic frequency by varying the time-coding sequences, thereby realizing the BFSK communication system.  Figure 11(a) illustrates the metasurface-based transmitter of the BFSK wireless communication system. Referring to [109] for details, the transmitting process mainly contains three steps: firstly, the FPGA baseband module generates a bit stream of the original information (e.g., a photo); subsequently, the bit stream is mapped to different sets of time-coding sequences, which can produce the specific harmonic distributions required by the BFSK scheme; and finally, the incident wave is modulated by the time-coding sequences and the TDC metasurface transmits the modulated EM waves carrying the digital information. The measured results are illustrated at the bottom of Figure 11(a), in which a picture is successfully recovered by a software-defined radio (SDR) receiver with a message transmission rate of 78.125 kbps at the frequency of 3.6 GHz.

To further improve the message transmission rate, a quadrature phase shift keying (QPSK) modulation scheme was proposed for a wireless communication system [95]. This QPSK wireless communication system can smoothly transmit a video with a message transmission rate of ~1.6 Mbps at 4 GHz.  Figure 11(b) shows the indoor scenario of the QPSK system for video transmission and the corresponding measured constellation diagrams according to different communication distances. Moreover, some high-order modulation schemes such as 8PSK and 16QAM were presented in [96]. By regulating the reflection phases of the TDC digital metasurface, arbitrary constellation diagrams can be synthesized at the +1^st^ harmonic frequency.  Figure 11(c) shows the block diagram of the communication transmitter based on the TDC digital metasurface and the constellation diagram of QPSK, 8PSK, and 16QAM. For experimental validation, a microwave prototype of the TDC digital metasurface working around the frequency of 4.25 GHz was fabricated, as shown in Figure 11(d).

Overall, numerical and experimental results demonstrate the good performance of the new architecture wireless communication systems, which significantly simplify the system architecture and have promising applications in the future 6G communication scenario.

### 3.10. Space- and Frequency-Division Multiplexing Wireless Communication Scheme

As previously mentioned, the TDC digital metasurfaces were effectively used to build the new architecture wireless communication systems, which support BFSK, QPSK, 8PSK, and 16QAM. However, space-domain modulation is not implemented in such systems [95–97, 109]. In fact, the TDC digital metasurfaces have the limitation of transmitting the same information to receivers at different spatial directions, in which case only the power levels of the received signals are different. Therefore, undesired users at other locations can still recover the correct information by using a sufficiently sensitive receiver. The STC digital metasurface can lift this restriction by also implementing information encoding in the space domain. On the other hand, multiplexing techniques have been widely used to establish multiple independent channels between transmitters and receivers, which can improve the capacity of communications. Some multiplexing techniques have been developed in the past decades, such as time-division multiplexing (TDM), frequency-division multiplexing (FDM), space-division multiplexing (SDM), and code-division multiplexing (CDM). FDM requires high-performance filters and mixers to divide the frequency range. SDM is usually realized by using a phased array, which is composed of many antennas and radiofrequency (RF) chains, leading to a communication system with high cost and high complexity.

**Figure 12:**
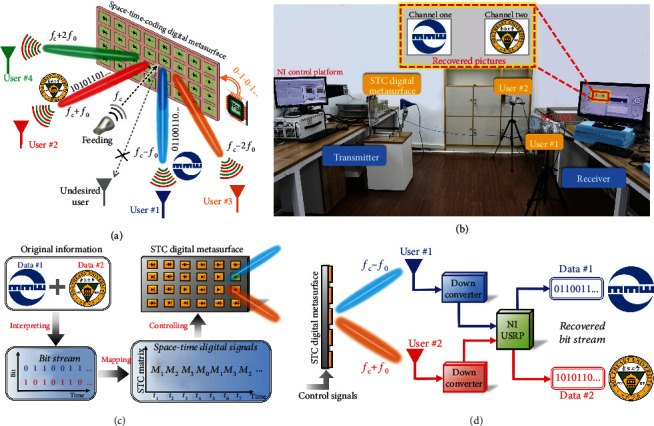
Space- and frequency-division multiplexing wireless communication scheme. Reproduced from [118]. (a) Conceptual illustration of multichannel direct data transmissions via an STC digital metasurface. (b) The testbed of the wireless communication system, in which two different colored pictures are transmitted from the transmitter (the left part) to two users (the right part) simultaneously and individually. Block diagram of (c) the transmitting process and (d) the receiving process.

The STC digital metasurfaces with simultaneous controls of the spatial and spectral characteristics can be used to overcome the aforementioned limitations and challenges, which have the advantages of low cost, simple structures, and easy fabrications. In [118], a new information encoding scheme based on the STC digital metasurface was proposed to implement both the space- and frequency-division multiplexing techniques in a multichannel wireless communication system. By encoding the optimized STC matrices (see Figure 2 in [118]), different digital messages can be directly transmitted to multiple users simultaneously and independently. As illustrated in Figure 12(a), a multichannel wireless communication system of direct data transmission is established by using an STC digital metasurface. In this case, different data streams are directly routed to designated users (e.g., Users #1, #2, #3, and #4) located in different directions. Each designated user has its own independent receiving channel via a specific frequency, while undesired users located in other directions cannot recover the correct information.

For experimental validation, a dual-channel wireless communication system based on a 2-bit STC digital metasurface was built to transmit two different pictures to two users, as shown in Figure 12(b). Referring to [118] for more details, the system transmitter mainly includes a control platform, a microwave signal generator, and the STC digital metasurface fed by a linearly polarized horn antenna, as shown in the left part of Figure 12(b). Accordingly, the receiving terminals mainly contain two horn antennas, down converters, the SDR receiver, and the postprocessing computer, as displayed in the right part of Figure 12(b). The process of dual-channel direct data transmissions was detailed in [118]. Figures 12(c) and 12(d) show the transmitting and receiving processes, respectively. Measured results show that two different pictures are directly transmitted to two users simultaneously in real time with a high transmission rate (see the inset in Figure 12(b)), validating the feasibility of the space- and frequency-multiplexed information encoding scheme. More results of the dual-channel system with arbitrary user locations as well as the three-channel system can be found in [118].

The STC digital metasurface simultaneously plays the role of information modulation and energy radiation. The STC strategy provides a low-cost solution for realizing SDM and FDM wireless communication, eliminating the requirement for antenna arrays, filters, and mixers. This new wireless communication scheme based on STC digital metasurfaces has low interference between different user channels and also offers the characteristic of directional modulation for secure transmission, which has important applications in secure communications, multicarrier communications, frequency-hopping communications, and future radar systems.

## 4. Conclusions and Perspectives

In conclusion, the STC digital metasurfaces provide versatile and powerful platforms for implementing spatiotemporal modulations, which enable advanced EM field manipulations in both the space and frequency domains and open up a new degree of freedom to expand the functionality of information metasurface systems. Owing to the huge advantages of the STC strategy, wave manipulation and information processing can be simultaneously performed in a multidimensional domain, leading to numerous applications in the fields of electromagnetics and information science. In this review article, we have summarized the general concepts and working principles of STC digital metasurfaces and presented their recent progress and representative applications, focusing particularly on the harmonic beam steering/shaping, scattering-signature reduction, programmable nonreciprocal effect, arbitrary multibit programmable phases, nonlinear harmonic manipulations, frequency synthesis, polarization conversion, convolution operation, joint multifrequency syntheses, harmonic information transitions, and new architecture wireless communication systems.

Among the most interesting applications of the STC digital metasurfaces, it is worth mentioning the reconfigurable intelligent surfaces (RISs) [127–131], which are also widely referred to as intelligent reflecting surfaces (IRSs) [132, 133]. RISs are currently attracting a lot of attention for the next-generation 6G wireless communication networks. By deploying RISs in the wireless propagation environment, the wireless channels can be reprogrammable and controllable in time, leading to the smart radio environments [128, 133]. RISs help improve the signal-to-noise ratio (SNR) via beamforming [132] and achieve signal coverage enhancement or interference suppression at designated receivers. By installing the low-cost and energy-efficient RISs on building walls, transmitted signals will be reflected by the RISs and then propagated along designated directions by controlling the elements' phases and/or amplitudes, thereby establishing supplementary links for wireless communications and information/power transfer systems [128, 133]. Within this overarching framework of RIS-assisted wireless networks, the STC digital metasurfaces provide a possibility to remove the complex modulation/encoding and RF processing modules that are usually required in the traditional wireless communication systems and enable a simple implementation of diversity and multiplexing in terms of frequency, space, time, polarization, and pattern [118]. Other promising applications inspired by the STC digital metasurfaces include the next-generation information systems of cognitive radars, microwave computational imaging, smart sensing/recognition, and advanced mathematical operations. Besides, more information theory and signal processing methods can be explored to analyze the physical intentions behind STC coding metasurfaces, which is expected to provide guidance for future information systems.

However, there also exist some challenges that need to pay attention to. The current STC digital metasurfaces are experimentally realized with only phase modulations, where the amplitude and phase-amplitude modulations have not yet been achieved in practice. Thus, one important challenge is the joint phase-amplitude modulation scheme, which is difficult to realize in the practical design of the programmable metasurfaces but has great potential for improving the spectral efficiency of multiple harmonic frequencies [126]. In addition, the current reflection-type STC digital metasurface may encounter the issue of blockage caused by the feeding antenna in some specific scenarios. To avoid the blockage effects, one could design a transmission-type STC digital metasurface with the feeding antenna behind it [76] or a waveguide-fed metasurface architecture [134, 135].

Another critical aspect is about the modulation mechanism and switching speed of the programmable character. Most experimental realizations of STC digital metasurfaces to date are based on PIN or varactor diodes, which have limited switching speed and are restricted to the microwave frequency band. In some applications, such as the scenario of the nonreciprocal effect illustrated in Section 3.6, it is crucial to improve the modulation frequency (switching speed) for attaining enough angular separation in the space domain. So far, some faster switching schemes such as graphene [34, 136], vanadium dioxide [137, 138], and 2D electron gas [139] have been explored for phase/amplitude modulations in the metasurface applications to reach higher-frequency ranges (e.g., terahertz) [140], which are promising for implementing faster spatiotemporal modulation and paving the way for more advanced applications in the future.
